# Phylogenetic evidence for a clade of tick-associated trypanosomes

**DOI:** 10.1186/s13071-022-05622-y

**Published:** 2023-01-05

**Authors:** Rachid Koual, Marie Buysse, Justine Grillet, Florian Binetruy, Sofian Ouass, Hein Sprong, Maxime Duhayon, Nathalie Boulanger, Frédéric Jourdain, Aurélien Alafaci, Julien Verdon, Hélène Verheyden, Claude Rispe, Olivier Plantard, Olivier Duron

**Affiliations:** 1 MIVEGEC, CNRS, IRD, University of Montpellier, Montpellier, France; 2 Laboratory for Zoonoses and Environmental Microbiology (Z&O), Centre for Infectious Disease Control (CIb), National Institute of Public Health and Environment (RIVM), Bilthoven, The Netherlands; 3 ASTRE, CIRAD, INRAE, University of Montpellier, Montpellier, France; 4 UR7290: VBP: Borrelia Group, Hôpitaux Universitaires de Strasbourg, University of Strasbourg and French National Reference Center for Borrelia, Strasbourg, France; 5 UMR CNRS 7267, EBI, University of Poitiers, Poitiers, France; 6 INRAE, CEFS, Université de Toulouse, Castanet Tolosan Cedex, France; 7 LTSER ZA PYRénées GARonne, Auzeville-Tolosane, France; 8 Oniris, INRAE, BIOEPAR, Nantes, France

**Keywords:** *Trypanosoma pestanai*, Trypanosome specificity, *Amblyomma*, *Ixodes*

## Abstract

**Background:**

Trypanosomes are protozoan parasites of vertebrates that are of medical and veterinary concern. A variety of blood-feeding invertebrates have been identified as vectors, but the role of ticks in trypanosome transmission remains unclear.

**Methods:**

In this study, we undertook extensive molecular screening for the presence and genetic diversity of trypanosomes in field ticks.

**Results:**

Examination of 1089 specimens belonging to 28 tick species from Europe and South America led to the identification of two new trypanosome strains. The prevalence may be as high as 4% in tick species such as the castor bean tick *Ixodes ricinus*, but we found no evidence of transovarial transmission. Further phylogenetic analyses based on 18S rRNA, *EF1-α*, *hsp60* and *hsp85* gene sequences revealed that different tick species, originating from different continents, often harbour phylogenetically related trypanosome strains and species. Most tick-associated trypanosomes cluster in a monophyletic clade, the *Trypanosoma pestanai* clade, distinct from clades of trypanosomes associated with transmission by other blood-feeding invertebrates.

**Conclusions:**

These observations suggest that ticks may be specific arthropod hosts for trypanosomes of the *T. pestanai* clade. Phylogenetic analyses provide further evidence that ticks may transmit these trypanosomes to a diversity of mammal species (including placental and marsupial species) on most continents.

**Graphical Abstract:**

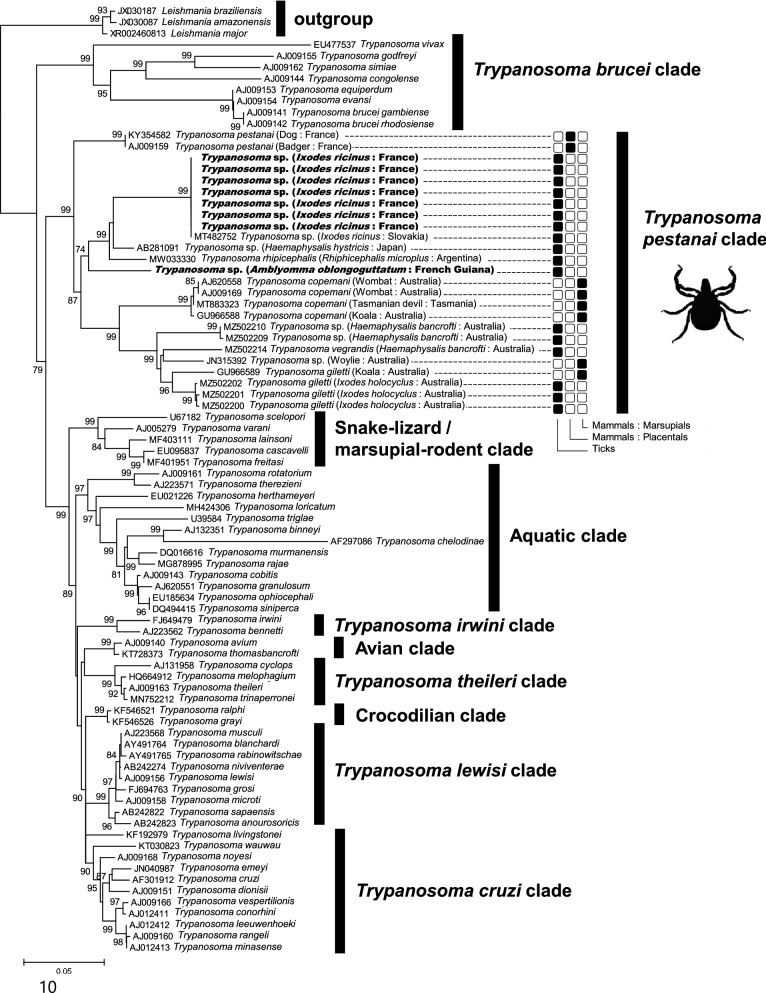

**Supplementary Information:**

The online version contains supplementary material available at 10.1186/s13071-022-05622-y.

## Background

Trypanosomes (Kinetoplastida, Trypanosomatidae, *Trypanosoma*) are vector-borne protozoan parasites that infect vertebrates [1–3]. Several trypanosome species are important human health concerns as the etiological agents of American trypanosomiasis (Chagas disease), *Trypanosoma cruzi* [4] and human African trypanosomiasis (sleeping sickness), *T. brucei rhodesiense* and *T. brucei gambiense* [5]. Some trypanosome species are also of concern to domestic animals, including the agents of African animal trypanosomiasis, such as *T. congolense* and *T. vivax*, and the agent of surra, *T. evansi*, which have a high economic burden [6]. Many other trypanosome species have been reported from wildlife, such as *T. pestanai*, primarily found in Eurasian badgers [7–9]. To date, a diversity of trypanosome species classified into at least 10 *Trypanosoma* infrageneric clades have been described from all vertebrate classes [2, 3, 10].

Vector specificity, in other words, the extent to which a parasite can be transmitted by different vector species, is a fundamental parameter for trypanosome life-cycles. Successful transmission to vertebrates is at least partly conditioned by trypanosome adaptation to vector physiology and behaviour [2, 3, 10]. For example, despite the ability of *T. cruzi* and *T. brucei* to infect humans, the former is transmitted only by triatomine bugs through the faecal (stercorarian) route [4], whereas the latter is transmitted by tsetse flies through saliva during biting [5]. Other examples include *T. lewisi* transmitted by fleas to rodents and *Trypanosoma* spp. belonging to the ‘lizard-snake/marsupial-rodent’ clade, which are all transmitted by sand flies [11]. Therefore, not all blood-feeding invertebrates are able to transmit all trypanosomes, and only restricted pairings of blood-feeding invertebrates and trypanosome species can successfully transmit infection to vertebrates [2, 3]. Exceptions exist, however, such as for *T. evansi*, which is mechanically transmitted mainly by hematophagous flies, and to a lesser extent by other animals (e.g., vampire bats) that have recently fed on parasitaemic vertebrates [6].

Ticks have long been proposed as vectors of trypanosomes, but their importance in transmission cycles remains unclear [12–16]. Early microscopic morphological observations provided evidence for the presence of trypanosomes in a variety of tick species [17–20]. More recently, molecular investigations led to the detection of trypanosomes from at least 15 tick species in Europe, Asia, Australia and South America (Table 1). Based on 18S rRNA gene sequences and morphological observations, two novel trypanosome species detected in ticks have been formally described in Brazil: *Trypanosoma rhipicephalis* isolated from the cattle tick *Rhipicephalus microplus* [16] and *T. amblyommi* from *Amblyomma brasiliense* [15]. The other trypanosomes found in ticks have not been formally named, but some belong to previously described species from mammals or are closely related to them (Table 1). Indeed, *Trypanosoma copemani*, *T. gilletti* and *T. vegrandis* were first described from Australian marsupials and further detected in Australian *Ixodes* ticks feeding on these vertebrate hosts [12, 21, 22]. These observations have drawn attention to the potential role of ticks as trypanosome vectors, but they are still not conclusive evidence of vector competence: ticks feeding on parasitaemic vertebrates will be positive for trypanosome DNA due to the presence of infected blood in the tick digestive tract, but may not be competent to transmit infectious trypanosomes during their next blood meal [13].

**Table 1:** List of tick species in which trypanosomes have been detected using molecular detection methods, including the present study

Geographical origin		Tick species	*Trypanosoma* species/strain	Tick feeding status	References
Europe	Slovakia	*Ixodes ricinus*	*Trypanosoma* sp. strain Bratislava 1	Questing (unfed) ticks	[14]
	France	*Ixodes ricinus*	*Trypanosoma* sp.	Questing (unfed) ticks, and ticks (engorged) collected on roe deer (*Capreolus capreolus*)	This study
	Italy	*Ixodes ricinus*	*Trypanosoma pestanai*	Ticks (engorged) collected on badgers (*Meles meles*)	[9]
	Italy	*Ixodes canisuga*	*Trypanosoma pestanai*	Ticks (engorged) collected on badgers (*Meles meles*)	[9]
Asia	Pakistan	*Hyalomma anatolicum*	*Trypanosoma* sp. strain t-PACA-88	Ticks (engorged) collected on cattle	[27]
	Japan	*Haemaphysalis flava*	*Trypanosoma* sp. strain 17ISK-T2, *Trypanosoma* sp. strain 17ISK-T22	Questing (unfed) ticks	[47]
	Japan	*Haemaphysalis hystricis*	*Trypanosoma* sp.	Questing (unfed) ticks	[48]
South America	Argentina	*Rhipicephalus microplus*	*Trypanosoma rhipicephalis* strain Chaco CB	Ticks (engorged) collected on cattle	[49]
	Brazil	*Rhipicephalus microplus*	*Trypanosoma rhipicephalis* strain P1RJ	Ticks (engorged) collected on cattle	[16]
	Brazil	*Amblyomma brasiliense*	*Trypanosoma amblyommi*	Ticks (engorged) collected on a white-lipped peccary (*Tayassu pecari*)	[16]
	French Guiana	*Amblyomma oblongoguttatum*	*Trypanosoma* sp.	Questing (unfed) ticks	This study
Oceania	Australia	*Ixodes australiensis*	*Trypanosoma copemani*, *T. vegrandis*	Ticks (engorged) collected on marsupial hosts	[12, 21]
	Australia	*Ixodes tasmani*	*Trypanosoma copemani*, *T. vegrandis*, *T. gilletti*, *T. irwini*, *Trypanosoma* sp. AB-2017	Ticks (engorged) collected on marsupial hosts	[12, 22]
	Australia	*Ixodes woyliei*	*Trypanosoma copemani*, *T. vegrandis*	Ticks (engorged) collected on marsupial hosts	[12]
	Australia	*Ixodes myrmecobii*	*Trypanosoma* sp.	Ticks (engorged) collected on marsupial hosts	[12]
	Australia	*Ixodes holocyclus*	*Trypanosoma copemani*, *T. gilletti*, *T. irwini*, *Trypanosoma* sp. AB-2017	Questing (unfed) ticks, and ticks (engorged) collected on koalas (*Phascolarctos cinereus*)	[21, 24]
	Australia	*Haemaphysalis bancrofti*	*Trypanosoma vegrandis*, *Trypanosoma* sp. nov. HB	Questing (unfed) ticks	[24]
	Australia	*Amblyomma triguttatum*	*Trypanosoma copemani*, *T. vegrandis*, *T. noyesi*	Questing (unfed) ticks, and ticks (engorged) collected on marsupial hosts	[12, 36]

Only a few studies have investigated the vector competence of ticks for trypanosomes [13]. Transmission to cattle by the bite of *Hyalomma anatolicum* ticks has been reported at least once [23]. Viable forms of *T. copemani* were also observed in faecal materials of *Ixodes australiensis* ticks, suggesting that transmission may be possible through the stercorarian route [21]. The molecular detection of trypanosomes from questing (unfed) ticks (e.g., *Ixodes ricinus*, *I. holocyclus*, *Amblyomma triguttatum* and *Haemaphysalis bancrofti*) that have already digested their previous blood meals, have further moulted (i.e., transstadial transmission), and are seeking vertebrates for their next blood meal further suggests that ecologically and taxonomically diverse ticks can acquire and maintain trypanosomes [12, 14, 24]. The in vitro isolation of viable trypanosomes from questing *I. ricinus* further corroborates this finding [14]. The recent observation of *T. rhipicephalis* DNA in some eggs obtained from artificially infected females of the brown dog tick *Rhipicephalus sanguineus* also suggests that trypanosomes may be transmitted by the transovarial route in ticks [25]. However, the role of ticks as vectors of trypanosomes remains controversial. Metacyclic trypanosomes, which are the infective forms transmissible to vertebrates, have not yet been observed in ticks, and trypanosomes are more often reported from engorged ticks collected on parasitaemic vertebrates than from questing ticks [13]. This lack of conclusive evidence emphasizes the current need for novel approaches to study the significance of ticks in trypanosome transmission cycles.

Here we approached this issue by undertaking extensive screening for the presence and diversity of trypanosomes in natural populations of 28 tick species from Europe (France and the Netherlands) and South America (French Guiana). We further conducted a phylogenetic analysis of the trypanosomes found in ticks to investigate their degree of specialization for ticks. To this aim, we estimated how phylogenetically related are trypanosomes shared among tick species in nature, including 18S rRNA (small subunit [SSU]), *EF1-α*, *hsp60* and *hsp85* gene sequences obtained in this study and those already available on public databases. If ticks are vectors of trypanosomes, we predict that they should harbour phylogenetically related trypanosomes clustering in a monophyletic clade distinct from other trypanosome clades associated with transmission by other blood-feeding invertebrates.

## Methods

### Tick collection

Field ticks (*n*=1089; belonging to 28 tick species) were collected in French Guiana (*n* = 652; 22 species) and Europe (*n* = 437; 6 species) either on vegetation by the drag-flag method (for questing ticks) or directly from their vertebrate hosts or seabird nests (for engorged ticks) (Table 2; Additional file 1: Table S1). Samples included five major tick genera belonging to the family Ixodidae (hard ticks): *Amblyomma* (16 species), *Ixodes* (5 species), *Dermacentor* (3 species), *Rhipicephalus* (2 species) and *Haemaphysalis* (1 species). Samples include one species of the *Ornithodoros* genus, belonging to the Argasidae family (soft ticks). All ticks, except gravid females, were preserved in 75% ethanol until examination. Gravid females (all from France) were captured alive from their vertebrate hosts. They were brought to the laboratory, individually housed in plastic cups and kept at 25 °C for egg laying. Females and eggs were further collected and preserved in 75% ethanol. All tick specimens were examined under a Leica Z16 APO A macroscope and categorized by species, stage and sex (Table 2; Additional file 1: Table S1) [26].

**Table 2:** List of tick species screened in this study for the presence of trypanosomes. See also Table S1 for more details. L, larva; N, nymph; M, male; F, female

Geographical origin	Tick species	No.	Stage (L, N, M, F)	No. of *Trypanosoma*-positive (%)
South America (French Guiana)				
	*Amblyomma cajennense* sensu stricto (Fabricius, 1787)	217	121N, 51M, 45F	0
	*Amblyomma calcaratum* Neumann, 1899	1	1L	0
	*Amblyomma coelebs* Neumann, 1899	14	14N	0
	*Amblyomma dissimile* Koch, 1884	22	2N, 12M, 8F	0
	*Amblyomma geayi* Neumann, 1899	10	8L, 2M	0
	*Amblyomma goeldii* Neumann, 1899	5	2M, 3F	0
	*Amblyomma humerale* Koch, 1844	10	4N, 5M, 1F	0
	*Amblyomma latepunctatum* Tonelli-Rondelli, 1939	4	4N	0
	*Amblyomma longirostre* (Koch, 1844)	136	134L, 6N	0
	*Amblyomma naponense* (Packard, 1869)	5	3N, 1M, 1F	0
	*Amblyomma oblongoguttatum* Koch, 1844	66	36M, 30F	1 (1.5%)
	*Amblyomma pacae* Aragão, 1911	5	5L	0
	*Amblyomma romitii* Tonelli-Rondelli, 1939	2	2F	0
	*Amblyomma rotundatum* Koch, 1844	6	5L, 1F	0
	*Amblyomma scalpturatum* Neumann, 1906	9	6N, 1M, 2F	0
	*Amblyomma varium* Koch, 1844	7	3L, 1N, 2M, 1F	0
	*Dermacentor nitens* Neumann, 1897	97	27L, 32N, 20M, 18F	0
	*Haemaphysalis juxtakochi* Cooley, 1946	8	8N	0
	*Ixodes luciae* Senevet, 1940	6	1L, 2N, 2M, 1F	0
	*Ornithodoros capensis* sensu stricto Neumann, 1901	6	2N, 2M, 2F	0
	*Rhipicephalus microplus* (Canestrini, 1888)	10	4L, 6M	0
	*Rhipicephalus sanguineus* sensu lato (Latreille, 1806)	6	3M, 3F	0
Europe (France and the Netherlands)				
	*Dermacentor marginatus* (Sulzer, 1776)	148	54M, 94F	0
	*Dermacentor reticulatus* (Fabricius, 1794)	86	32M, 54F	0
	*Ixodes frontalis* (Panzer, 1798)	11	4N, 7F	0
	*Ixodes hexagonus* Leach, 1815	4	4F	0
	*Ixodes ricinus* (Linnaeus, 1758)	178	26N, 61M, 91F	7 (3.9%)
	*Ixodes ventalloi* Gil Collado, 1936	10	10F	0

### Molecular detection and typing of trypanosomes

DNA was extracted from individual ticks using the DNeasy Blood & Tissue Kit (QIAGEN) following the manufacturer's instructions. Each individual extract was then tested by nested polymerase chain reaction (PCR) for *Trypanosoma* infection by amplifying a fragment of the 18S rRNA (SSU) gene (Additional file 1: Table S2).

Nested PCR amplifications were performed as follows: The first PCR run with the external primers was performed in a 10 μl volume containing 10–50 ng of genomic DNA, 3 mM of each deoxyribonucleoside triphosphate (dNTP) (Thermo Scientific), 8 mM of MgCl_2_ (Roche Diagnostics), 3 μM of each primer, 1 μl of 10× PCR buffer (Roche Diagnostics) and 0.5 U of Taq DNA polymerase (Roche Diagnostics). A 1 μl aliquot of the PCR product from the first reaction was then used as a template for the second round of amplification. The second PCR was performed in a total volume of 25 μl containing 8 mM of each dNTP (Thermo Scientific), 10 mM of MgCl_2_ (Thermo Scientific), 7.5 μM of each of the internal primers, 2.5 μl of 10× PCR buffer (Thermo Scientific) and 1.25 U of Taq DNA polymerase (Thermo Scientific). All PCR amplifications were performed as follows: initial denaturation at 93 °C for 3 min, 35 cycles of denaturation (93 °C, 30 s), annealing (*T*_m_ = 52 °C) and extension (72 °C, 1 min), and a final extension at 72 °C for 5 min. Positive (DNA of a trypanosome-infected sloth) and negative (water) controls were included in each PCR assay. The PCR products were electrophoresed in a 1.5% agarose gel. All positive PCR products were purified and sequenced (Eurofins Genomics) in both directions to ensure that the record represented a true positive and not a PCR artefact or related protozoa. Sequence chromatograms were manually cleaned with Chromas Lite (http://www.technelysium.com.au/chromas_lite.html), and alignments were performed using ClustalW (http://www.clustal.org/clustal2/), implemented in the MEGA software (https://www.megasoftware.net/).

After initial screening, additional PCR amplifications were conducted on all positive DNA samples to acquire larger 18S rRNA gene sequences for the *Trypanosoma* intrageneric phylogeny. To this aim, we amplified, sequenced and trimmed two 18S rRNA gene fragments for each sample to obtain 1900–2100-base pair (bp) trypanosome 18S rRNA gene sequences (Additional file 1: Table S2). We further amplified and sequenced trypanosome *EF1-α*, *hsp60* and *hsp85* gene fragments from positive DNA samples through nested or semi-nested PCR assays. Gene features and primers are listed in Additional file 1: Table S2. All PCR products were processed as described above.

### Phylogenetic analyses

We reconstructed the phylogenetic relationships between trypanosome species and strains using 18S rRNA, *EF1-α*, *hsp60* and *hsp85* gene sequences obtained in this study and sequences available in GenBank, including sequences of representative trypanosome species and sequences obtained from previous studies based on DNA extracted from ticks (Table 1). The 18S rRNA global dataset used for phylogenetic reconstructions included sequences from 59 trypanosome species, and sequences of three *Leishmania* species used as outgroups. Because previous studies of trypanosomes in ticks (listed in Table 1) have used different 18S rRNA typing protocols, some of the sequences available in GenBank were obtained from distinct positions along the 18S rRNA gene sequences. Indeed, while some studies sequenced fragments located near the 5′ end of the 18S rRNA gene sequence [12], other studies sequenced fragments rather located in the middle [27] or near the 3′ end [22]. In addition, most of these sequences are quite short (typically <350 bp) compared to the complete 18S rRNA gene sequence, which is 2100–2300-bp long depending on the trypanosome species [12, 22, 27]. As a result, most of these short 18S rRNA gene sequences cannot be used in the same phylogenetic analysis because they do not overlap and cannot be aligned with each other. To circumvent this issue and include all trypanosome sequences of ticks in our phylogenetic analyses, we thus created four separate datasets: The first dataset is an alignment of a large 18S rRNA gene fragment (1950 bp) and the three others are alignments based on distinct fragments of the 18S rRNA gene sequence (5′ end region: 240 bp; middle region: 540 bp; 3′ end region: 250 bp). Because 18S rRNA gene sequence has been commonly used as an exclusive taxonomic marker for the trypanosomes detected in ticks (cf. references in Table 1), no *EF1-α*, *hsp60* or *hsp85* gene sequences of tick trypanosomes were available on public databases. Phylogenetic investigations were thus based on *EF1-α*, *hsp60* and *hsp85* gene sequences produced in this study and those of representative trypanosome species available on GenBank.

For each dataset, the gBlocks program [28] was used to remove poorly aligned positions and to obtain unambiguous gene sequence alignments. The 18S rRNA phylogenies were based on nucleotide sequences while the *EF1-α*, *hsp60* and *hsp85* phylogenies were based on amino acid sequences because of ambiguities in their nucleotide alignments. Phylogenetic reconstructions were based on maximum likelihood (ML) analyses using the MEGA program (https://www.megasoftware.net/). The evolutionary models most closely fitting the sequence data were determined using Akaike information criterion. ML heuristic searches using starting trees obtained by neighbour-joining were conducted. The clade robustness of ML phylogenetic trees was further assessed by bootstrap analyses using 1000 replicates.

### Statistical analyses

**Fig. 1:**
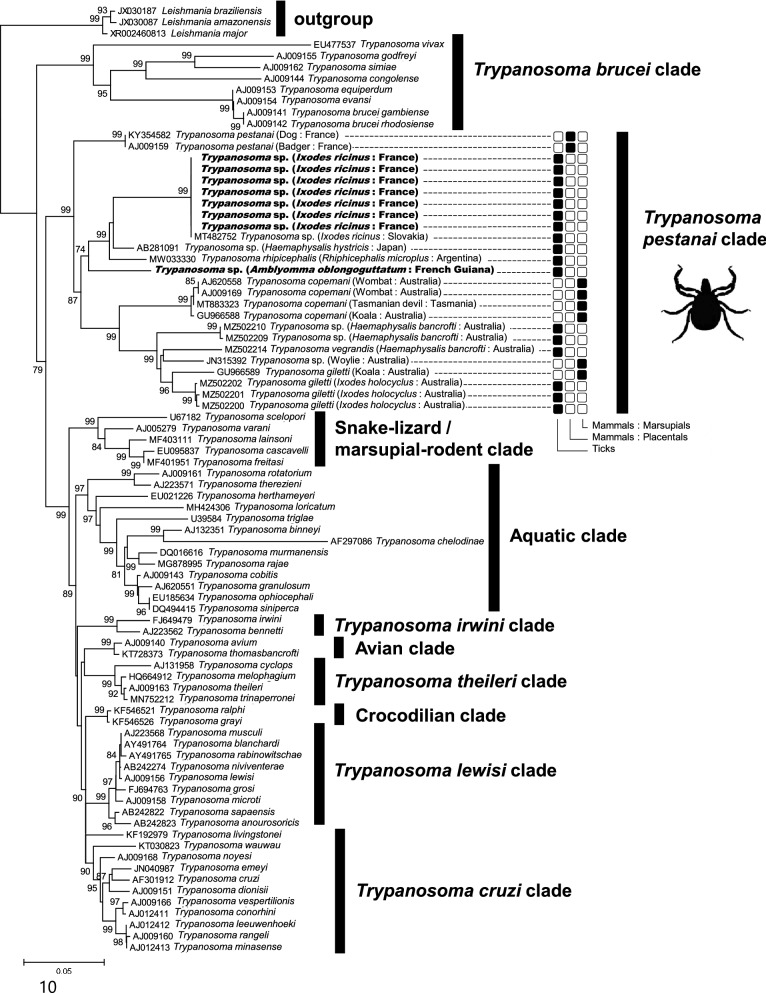
*Trypanosoma* phylogeny constructed using maximum likelihood (ML) estimations based on the 18S rRNA gene sequence (1305 unambiguously aligned bp; best-fit approximation for the evolutionary model: general time-reversible [GTR]) including *Trypanosoma* sequences obtained in this study (in bold). The 10 major *Trypanosoma* clades are indicated [2]. For members of the *T. pestanai* clade, origin of the *Trypanosoma* sequences (i.e., from ticks, mammals either marsupials or placentals) is shown by black squares, while host species and geographical origin are listed next to the name of *Trypanosoma* species or strain. GenBank accession numbers of sequences used in analyses are shown on the phylogenetic trees. Numbers at nodes indicate percentage support of 1000 bootstrap replicates. Only bootstrap values >70% are shown

All statistical analyses were carried out using the R statistical package (https://www.r-project.org/). We tested for vector specificity by investigating the association of the phylogenetic distribution of trypanosomes with ticks (trypanosomes found in ticks vs all other trypanosomes). To this aim, we used the *D* metric [29] to estimate the overall degree of clustering of trypanosomes found in ticks in the trypanosome phylogeny (based on the alignment of the largest 18S rRNA gene fragment; Fig. 1). If trypanosomes detected in ticks are not randomly distributed over the phylogeny, it may be assumed that they are more phylogenetically clustered than expected by chance, and thus indicate a preferential association with ticks (that is, a specialization for ticks). The *D* metric provides an estimate of phylogenetic conservatism for binary traits that can be compared with a random shuffle of trait values at the tips of a phylogeny [29]. A *D* value of 1 indicates a phylogenetically random distribution, whereas a *D* value inferior to 1 indicates phylogenetic clustering. We further assessed the significance of the D metric estimates using permutation tests: *P *(*D* < 1) indicates whether the *D* metric is significantly smaller than 1, meaning that the trait is not randomly distributed over the phylogeny. We calculated the *D* metric implemented by the function ‘phylo.d’ in the R package *caper* with the default parameter of 1000 permutations.

## Results

### Detection of trypanosomes in ticks

Of the 1089 specimens, 18S rRNA-nested PCR assay indicated the presence of trypanosomes in eight ticks (0.7%). Of the 28 tick species, two species included at least one infected specimen: *Amblyomma oblongoguttatum* (*n* = 1) in one locality in French Guiana and *Ixodes ricinus* (*n* = 7) in three localities in France (Table 2; Additional file 1: Table S1). The detection rate of trypanosomes co-varied with the screening effort, i.e., the number of examined specimens per tick species (Spearman's rank correlation, *n* = 28, *r*_s_ = 0.39, *P* = 0.03): The tick species observed with trypanosomes were among those for which we examined more specimens. This was shown by *A. oblongoguttatum* (*n* = 66 examined specimens, one trypanosome-positive specimen: 1.5%) and *I. ricinus* (*n* = 178 examined specimens, seven trypanosome-positive specimens: 3.9%). However, there were also trypanosome-negative tick species for which we examined a large number of specimens, such as *A. longirostre* (*n* = 136 examined specimens but none positive) and *Dermacentor nitens* (*n* = 97 examined specimens but none positive) (Table 2; Additional file 1: Table S1). Of the eight trypanosome-infected tick specimens, two (one each of *A. oblongoguttatum* and *I. ricinus*) were questing ticks, and six (*I. ricinus*) were engorged ticks collected while feeding on roe deer.

Of the 1089 specimens, 62 were gravid females collected in France and belonging to six tick species, including *D. marginatus* (*n* = 4 females), *D. reticulatus* (*n* = 1), *I. frontalis* (*n* = 4), *I. hexagonus* (*n* = 3), *I. ricinus* (*n* = 43) and *I. ventalloi* (*n* = 7). Among these females, four *I. ricinus* females were found infected by trypanosomes. No evidence of maternal transmission was found: We examined individually 10 randomly sampled eggs per infected female (total of examined eggs = 40) using the 18S rRNA-nested PCR assay and never detected the presence of trypanosomes.

Molecular typing of trypanosomes

Based on 18S rRNA sequencing, two distinct trypanosomes were characterized: one was found in the *A. oblongoguttatum*-infected specimen, while the other was found in the seven *I. ricinus*-infected specimens (94.9% nucleotide identity). No nucleotide variation was observed between the trypanosome 18S rRNA gene sequences obtained from seven *I. ricinus*-infected specimens. When compared to already described trypanosome species, the trypanosomes of *A. oblongoguttatum* and *I. ricinus* showed the highest 18S rRNA pairwise nucleotide identities with *T. pestanai* (94.3% and 93.5%, respectively; GenBank accession number: AJ009159) and *T. copemani* (93.8% and 92.6%, respectively; GenBank accession number: AJ620558). However, the trypanosome of *I. ricinus* was 99.9% identical to the *Trypanosoma* sp. strain Bratislava 1 (GenBank accession number: MT482752) recently found in *I. ricinus* from Slovakia [14]. Although we collected some infected *I. ricinus* feeding on roe deer, the trypanosome strain detected in our ticks was clearly distinct from the trypanosomes previously identified in this mammal (pairwise nucleotide identities <84%; GenBank accession numbers: KJ397593–KJ397597, KF805972, HM138533); the trypanosomes of roe deer are considered to belong to the species *T. theileri* [30].

We further obtained the trypanosome *EF1-α*, *hsp60* and *hsp85* gene sequences from the seven infected *I. ricinus* specimens, and the *hsp60* and *hsp85* gene sequences from the infected *A. oblongoguttatum* specimen (no *EF1-α* PCR product was obtained from this latter). Based on the nucleotide sequences of *EF1-α*, *hsp60* and *hsp85*, no variation was observed between seven trypanosome-positive *I. ricinus* specimens. The *hsp60* and *hsp85* nucleotide sequences of these seven trypanosome-positive *I. ricinus* specimens showed high nucleotide identities (81.7% and 85.1%) with the trypanosome of *A. oblongoguttatum*.

### Phylogeny of *Trypanosoma*

Initial phylogenetic analyses based on the 1950-bp 18S rRNA gene sequence alignment revealed a robust clustering of trypanosome species in 10 distinct *Trypanosoma* clades (Fig. 1), as previously reported [2, 10]: The *T. brucei* clade, including the agents of sleeping sickness transmitted by tsetse flies; the *T. pestanai* clade, represented by *T. pestanai* infecting Eurasian badgers and Australian *Trypanosoma* spp. infecting marsupials such as *T. copemani*, *T. vegrandis* and *T. gilletti*; the snake-lizard/marsupial-rodent clade; the aquatic clade, including *Trypanosoma* species from fishes, anurans and platypus; the avian clade; the *T. theileri* clade, with the nominal species corresponding to a cattle parasite of worldwide distributed; the *T. lewisi* clade, found mostly in rodents; the *T. cruzi* clade, comprising mammalian trypanosomes with worldwide distribution; the *T. irwini* clade; and the crocodilian clade, harbouring trypanosomes of crocodilians and alligators.

**Fig. 2:**
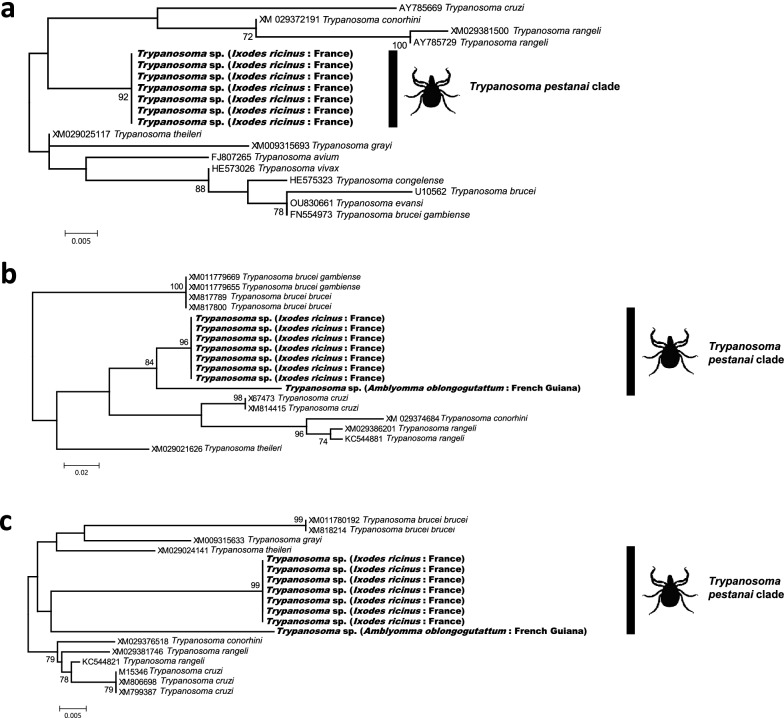
Phylogenies of *Trypanosoma* constructed using maximum likelihood (ML) estimations based on (**a**) *EF1-α* amino acid gene sequences (171 unambiguously aligned amino acid; best-fit approximation for the evolutionary model: Jones–Taylor–Thornton [JTT] + G), (**b**) *hsp60* amino acid gene sequences (142 unambiguously aligned amino acid; best-fit approximation for the evolutionary model: LG + G), (**c**) *hsp85* amino acid gene sequences (203 unambiguously aligned amino acid; best-fit approximation for the evolutionary model: LG + G). *Trypanosoma* sequences obtained from ticks in this study are shown in bold. GenBank accession numbers of sequences used in analyses are shown on the phylogenetic trees. Numbers at nodes indicate percentage support of 1000 bootstrap replicates. Only bootstrap values >70% are shown

Based on the 1950-bp 18S rRNA gene sequence alignment, the trypanosomes of *A. oblongoguttatum* and *I. ricinus* cluster in the *T. pestanai* clade with other trypanosomes detected in ticks, including *I. ricinus* from Slovakia, *I. holocyclus* from Australia, *R. microplus* from Argentina, *Haemaphysalis hystricis* from Japan and *Hae. bancrofti* (Fig. 1). The trypanosomes detected in ticks are not randomly distributed over the phylogeny. The association of these trypanosomes with ticks showed a significant phylogenetic signal (*D* = −0.69, *P *(*D* < 1) = 0.001), meaning that the trypanosomes of *A. oblongoguttatum*, *I. ricinus*, *I. holocyclus*, *R. microplus*, *Hae. hystricis* and *Hae. bancrofti* are more phylogenetically clustered than expected by chance. This phylogenetic clustering cannot be explained by geography. These tick species originate from different countries (*A. oblongoguttatum*: French Guiana; *R. microplus*: Brazil; *I. ricinus*: France; *Hae. hystricis*: Japan; *I. holocyclus* and *Hae. bancrofti*: Australia), and most of them have non-overlapping distribution ranges. No 18S rRNA gene sequence of a member of the *T. pestanai* clade obtained from blood-feeding invertebrates other than ticks is present in public databases. However, within the *T. pestanai* clade, the trypanosomes of ticks also cluster with trypanosomes infecting mammals, including *T. pestanai* infecting European badgers and dogs, and *T. copemani* and *T. gilletti*, infecting a diversity of Australian and Tasmanian marsupials (Figs. 1, 2A–C). *Trypanosoma copemani*, *T. gilletti* and *T. vegrandis* are distinct from the species *T. pestanai*, but all four have previously been found to be phylogenetically related species belonging to the *T. pestanai* clade [2, 10, 31–34]. Based on the 1950-bp 18S rRNA gene sequence alignment, *T. pestanai*, *T. copemani*, *T. gilletti* and *T. vegrandis* formed a robust clade, supported by a 99% bootstrap value, together with trypanosomes detected in ticks (Fig. 1).

Further phylogenetic reconstructions based on three distinct regions of the 18S rRNA gene sequence enabled extension of the analyses to include additional members of the *T. pestanai* clade (Additional file 1: Figs. S1–S3), including *T. caninum* infecting South American dogs [35], and *T. vegrandis* infecting a range of Australian marsupials [32]. Although the phylogenetic trees show some polytomies due to insufficient sequence polymorphism, each dataset confirmed the phylogenetic proximity of trypanosomes of *A. oblongoguttatum* and *I. ricinus* with other trypanosomes found in ticks. Also included in the *T. pestanai* clade are the trypanosomes found in 13 other tick species: *Hae. hystricis*, *Hae. falva*, *Hae. bancrofti*, *R. microplus*, *Hya. anatolicum*, *Amblyomma triguttatum*, *A. brasiliense*, *Ixodes canisuga*, *I. australiensis*, *I. woyliei*, *I. myrmecobii*, *I. holocyclus* and *I. tasmani* (Additional file 1: Figs. S1–S3, also listed in Table 1). Some of the trypanosomes found in ticks belong to already described species: *T. rhipicephalis* in *R. microplus*; *T. amblyommi* in *A. brasiliense*; *T. pestanai* in *I. ricinus* and *I. canisuga*; *T. vegrandis* in *A. triguttatum, Hae. bancrofti*, *I. australiensis*, *I. tasmani* and *I. woyliei*; *T. copemani* in *A. triguttatum, I. australiensis*, *I. tasmani*, *I. woyliei* and *I. holocyclus*; *T. gilletti* in *I. tasmani* and *I. holocyclus*. Some tick species harbour genetically distinct trypanosomes belonging to the *T. pestanai* clade, including *I. ricinus* with *T. pestanai* (in Italy) and *Trypanosoma* sp. (France and Slovakia), and *I. tasmani* with *T. vegrandis*, *T. copemani* and *T. gilletti* (Australia) (Additional file 1: Figs. S1–S3). Overall, tick species infected with trypanosomes belonging to the *T. pestanai* clade originate from four continents: Europe (*I. ricinus*, *I. canisuga*), Asia (*Hae. falva*, *Hae. hystricis*, *Hy. anatolicum*), South America (*A. oblongoguttatum*, *A. brasiliense*, *R. microplus*) and Australia (*I. australiensis*, *I. tasmani*, *I. woyliei*, *I. myrmecobii*, *I. holocyclus*, *I. tasmani*, *Hae. bancrofti*, *A. triguttatum*) (Additional file 1: Figs. S1–S3).

Based on the middle region of the 18S rRNA gene sequence, three tick species also harbour trypanosomes that do not fall within the *T. pestanai* clade: *A. triguttatum* with *T. noyesi* (belonging to the *T. cruzi* clade; Additional file 1: Fig. S4) [36], *I. tasmani* and *I. holocyclus* with *T. irwini* (*T. irwini* clade; Additional file 1: Fig. S5) [22]. However, while *T. noyesi* was identified in questing ticks [36], *T. irwini* was detected in engorged ticks from Koalas [22].

Further phylogenetic analyses based on *EF1-α*, *hsp60* and *hsp85* gene sequences confirmed the positioning of the trypanosomes of *A. oblongoguttatum* and *I. ricinus* within the *Trypanosoma* genus (Fig. 2A–C). The *hsp60* and *hsp85* phylogenetic trees further corroborated the close relatedness of the trypanosomes of *A. oblongoguttatum* and *I. ricinus* (no trypanosome *EF1-α* PCR product was obtained from *A. oblongoguttatum*; Fig. 2B, C). Few trypanosome *EF1-α*, *hsp60* and *hsp85* gene sequences were available on GenBank, including sequences mostly from trypanosomes of the *T. brucei* and *T. cruzi* clades but not from the *T. pestanai*, *T. caninum*, *T. vegrandis*, *T. copemani* and *T. gilletti* species. This lack of data made it impossible to compare the trypanosomes of *A. oblongoguttatum* and *I. ricinus* for these gene sequences with their putative closest relatives in the *T. pestanai* clade. However, *EF1-α*, *hsp60* and *hsp85* phylogenies consistently showed that the trypanosomes of *A. oblongoguttatum* and *I. ricinus* are distantly related to trypanosomes belonging to the *T. brucei*, *T. cruzi*, *T. theileri*, avian (with *T. avium*) and crocodilian (*T. grayi*) clades (Fig. 2B, C).

## Discussion

We show here that a wide diversity of trypanosomes is present in ticks. We conducted an extensive molecular screening of 28 tick species, and identified two distinct trypanosomes in a European tick species, *I. ricinus*, and a South American tick species, *A. oblongoguttatum*. No trypanosome was detected in the 26 other tick species. Combining our study with the current literature (Table 1) indicates that there is molecular evidence of trypanosome infections for at least 15 tick species in Europe, Asia, South America and Australia. Phylogenetic analyses further assign almost all of the trypanosomes found in these 15 tick species to the *T. pestanai* clade. Overall, these data converge to support the hypothesis that ticks are candidates for vectors of trypanosomes of the *T. pestanai* clade for a variety of mammals.

Four complementary lines of argument indicate a close association of members of the *T. pestanai* clade with ticks, which is suggestive of their vector specificity. The first is the phylogenetic relatedness of most *Trypanosoma* harboured by ticks in nature. They cluster in the *T. pestanai* clade, a pattern suggestive of the specialization of this phylogenetic clade of *Trypanosoma* for ticks. The second line of evidence concerns the extensive diversity of tick species and genera associated with members of the *T. pestanai* clade, as illustrated by the detection of infections in most major ixodid genera, including *Ixodes*, *Amblyomma*, *Rhipicephalus*, *Haemaphysalis* and *Hyalomma*, but not *Dermacentor*. This pattern is similar to the vector specificity observed for other *Trypanosoma* clades or species, such as *T. cruzi*, which can be transmitted by *ca.* 20 triatomine species belonging to different genera [4]. The third line of evidence is the broad geographical distribution of *Trypanosoma* associated with ticks. While the presence of *Trypanosoma* is rarely investigated in ticks, they have been detected from distant continents (Europe, Asia, South America and Australia), suggesting a worldwide distribution of the *T. pestanai* clade. However, the distribution of *T. pestanai* clade members is more structured geographically and fits with the distribution areas of tick species, as best shown with *T. pestanai* for European *Ixodes* species such as *I. ricinus* and *I. canisuga* [9], or with *T. vegrandis*, *T. copemani* and *T. gilletti* for Australian *Ixodes* and *Amblyomma* species [12, 21, 22]. Finally, phylogenies based on sequences available in public databases showed that no *Trypanosoma* was detected in blood-feeding invertebrates other than ticks cluster in the *T. pestanai* clade. However, ticks may not be the only vectors of members of the *T. pestanai* clade since fleas collected from a parasitaemic Eurasian badger were also found infected with *T. pestanai*, although vector competence of fleas has not been established [37]. This collective evidence suggests that ticks are potential vectors of *T. pestanai* clade members.

Beyond members of the *T. pestanai* clade, ticks have been reported in two instances to harbour more distantly related *Trypanosoma* species, at least in Australia [22, 36]. These *Trypanosoma* species, *T. irwini* and *T. noyesi*, have recently been described from Australian marsupials [34, 36, 38, 39]. *Trypanosoma noyesi* was further identified in questing ticks [36], but not *T. irwini*, which was only detected in engorged ticks [22], suggesting that there could be no reliable association between *T. irwini* and ticks. While *T. irwini* and *T. noyesi* have been found in *A. triguttatum* and *Ixodes* spp., they are less frequently detected in these Australian ticks than *T. vegrandis*, *T. copemani* and *T. gilletti* [12, 21, 22, 36]. The vectors of *T. noyesi* and *T. irwini* are currently unknown but tabanid and biting midges are the best vector candidates to date [38]. Overall, this means that infections by *Trypanosoma* species other than members of the *T. pestanai* clade may be only incidental in ticks, especially outside Australia.

Assuming ticks to be vectors of the *T. pestanai* clade implies that these trypanosomes are well adapted to tick physiology, life-cycle and behaviour, which raises the question of their mode of transmission. Mammalian trypanosomes are traditionally subdivided into two groups that follow distinct transmission modes: salivarian (through bite) and stercorarian (through faeces). Little evidence has been published on the mode of transmission by ticks, including salivarian [23] and stercorarian [21] transmission, and no definite conclusion can be drawn. It is worth noting that most phylogenetic analyses place the *T. pestanai* clade as a relative of a major salivarian clade, the *T. brucei* clade [2, 31–33, 35]. However, molecular phylogenetics consistently confirmed that salivarian and stercorarian *Trypanosoma* do not represent monophyletic groups, and therefore transmission mode cannot be inferred with certainty from phylogenetic positioning [2, 10, 33]. Alternatively, other transmission modes may exist since tick-borne protozoa belonging to the genus *Hepatozoon* are transmitted to vertebrates via ingestion of infected ticks [40]. Members of the *T. pestanai* clade might also be infectious via oral transmission, i.e., when a vertebrate eats an infected tick, but this remains to be investigated. Possible transovarial transmission was reported once for *T. rhipicephalis* in artificially infected *R. sanguineus* [25], but we did not observe evidence of maternal transmission in *I. ricinus*, suggesting that this transmission route may be a biological trait variable between members of the *T. pestanai* clade. Transstadial transmission for members of the *T. pestanai* clade is probable at least in *I. ricinus* and *A. oblongoguttatum* since we detected infections in unfed ticks (meaning that they have digested their previous blood meals and have further moulted), a pattern also previously observed in *I. ricinus*, *I. holocyclus*, *A. triguttatum* and *Haemaphysalis bancrofti* [12, 14, 24].

The risk of acquiring a tick-borne trypanosome infection is currently unknown, but the detection of members of the *T. pestanai* clade in ticks of significant medical or veterinary interest is of concern. Indeed, while *I. ricinus* is the tick species most commonly biting humans in Western Europe [41], in the present study, we found that 4% of field specimens were infected with a trypanosome of the *T. pestanai* clade. In Australia, a significant human-biting tick, *I. holocyclus*, was frequently found infected by *T. giletti* [24]. Humans are therefore exposed to bites of *Trypanosoma*-infected ticks without the risk of infection being documented to date. Domestic animals can readily be infected since infections with *T. pestanai* and *T. caninum* have been reported in dogs in Germany and Brazil, respectively [35, 42–44]. However, there are no clear symptoms of infection in dogs: *T. caninum* was detected in apparently healthy animals, suggesting that infection could be asymptomatic [44]; *T. pestanai* was detected in co-infection with another tick-borne pathogen (*Anaplasma phagocytophilum*) in a dog with malaise, pale mucous membranes and stiff joints, without knowing to which pathogen the symptoms could be attributed [43]. Another member of the *T. pestanai* clade, *T. rhipicephalis*, was isolated from engorged *R. microplus* specimens obtained from naturally infested cattle, but the effect of infection on cattle was not documented [16]. Some wild mammal species can also be infected and act as reservoir hosts for members of the *T. pestanai* clade. In Australia and Tasmania, endangered marsupials are consistently found infected by *T. vegrandis*, *T. copemani* and *T. gilletti*, raising the question of the importance of *Trypanosoma* infection for marsupial health and conservation [13, 34, 45, 46]. In Europe, badgers are commonly infected by *T. pestanai* with high prevalence [7–9]. Although we have collected infected ticks engorging on roe deer, the role of this mammal as reservoir for members of the *T. pestanai* clade seems unlikely since it is known to be rather infected by *T. theileri* [30]. Further studies are now necessary to investigate the pathogenicity, epidemiology, developmental cycles and transmission mechanisms of tick-borne trypanosomes.

## Conclusions

Ticks transmit a wider range of infectious agents than any other arthropod vector, but their role as vectors of trypanosomes remains less well-documented [13, 14]. Here, we used comparative phylogenetic methods to investigate whether ticks are infected by more closely related *Trypanosoma* species and strains than expected by chance, and then tested indirectly for arthropod host specificity. Our results indicate that ticks are potential vectors of members of the *T. pestanai* clade but not of other *Trypanosoma* clades or species, suggesting specific mechanisms regulating infection and transmission of members of the *T. pestanai* clade by ticks. Future work will require the use of an experimental approach to definitively demonstrate tick vector competence for trypanosome and to identify the transmission route (salivarian/stercorarian/transovarial) as well as the role ticks play in the biology of members of the *T. pestanai* clade.

## Supplementary Information

**Additional file 1**: **Table S1.** List of tick species screened in this study for the presence of trypanosomes. **Table S2.** Primers used in polymerase chain reaction (PCR) assays for trypanosomes screening survey and molecular typing. **Figure S1.** Phylogenies of the *Trypanosoma pestanai* clade constructed using the 5′ end region of the 18S rRNA gene sequence. **Figure S2.** Phylogenies of the *Trypanosoma pestanai* clade constructed using the middle region of the 18S rRNA gene sequence. **Figure S3.** Phylogenies of the *Trypanosoma pestanai* clade constructed using the 3′ end region of the 18S rRNA gene sequence. **Figure S4.** Phylogenies of trypanosomes including *Trypanosoma noyesi* of ticks and constructed using 18S rRNA gene sequences. **Figure S5.** Phylogenies of trypanosomes including *Trypanosoma irwini* of ticks and constructed using 18S rRNA gene sequences.

## Data Availability

Nucleotide sequences of the trypanosome 18S rRNA, *EF1-α*, *hsp60* and *hsp85* genes are available in the GenBank database under the following accession numbers: OQ025404-OQ025411 and OQ032675-OQ032697.

## Abbreviations

PCR: Polymerase chain reaction
ML: Maximum likelihood
