# Supplementary material for: Phylogenetic evidence for a clade of tick-associated trypanosomes
Source: Parasit Vectors. 2023 Jan 5;16:3. doi: 10.1186/s13071-022-05622-y (PMC9817367; doi:10.1186/s13071-022-05622-y)
Supplement: Supplementary file 1 — Additional file 1: Table S1. List of tick species screened in this study for the presence of trypanosomes. Table S2. Primers used in polymerase chain reaction (PCR) assays for trypanosomes screening survey and molecular typing. Figure S1. Phylogenies of the Trypanosoma pestanai clade constructed using the 5′ end region of the 18S rRNA gene sequence. Figure S2. Phylogenies of the Trypanosoma pestanai clade constructed using the middle region of the 18S rRNA gene sequence. Figure S3. Phylogenies of the Trypanosoma pestanai clade constructed using the 3′ end region of the 18S rRNA gene sequence. Figure S4. Phylogenies of trypanosomes including Trypanosoma noyesi of ticks and constructed using 18S rRNA gene sequences. Figure S5. Phylogenies of trypanosomes including Trypanosoma irwini of ticks and constructed using 18S rRNA gene sequences. [file 13071_2022_5622_MOESM1_ESM.pdf]

SUPPLEMENTARY DATA

Phylogenetic evidence for a clade of tick-associated trypanosomes

Koual et al.

Table S1 List of tick species screened in this study for the presence of trypanosomes. L, larva; N, nymph; M, male; F, female.

Ticks Species		Locality	Questing ticks / Engorged ticks (vertebrate hosts)	n	Stage (L, N, M, F)	*n Trypanosoma* -positive (%)
South America (French Guiana)						
1	*Amblyomma cajennense* sensus stricto (Fabricius, 1787)	Matoury, Piste de la Mirande, 2016-2017, French Guiana	Questing ticks	65	29N, 21M, 15F	0
		Kourou, Centre Spatial Guyanais, 2016, French Guiana	Questing ticks	72	12N, 30M, 30F	0
		Kourou, Montagne des Singes, 2016, French Guiana	Questing ticks	8	8N	0
		Petit-Saut, Montagne Plomb, 2016, French Guiana	Questing ticks	21	21N	0
		Kaw, Montagne de Kaw, 2016, French Guiana	Questing ticks	21	21N	0
		Sinnamary, Piste Saint-Elie, 2016, French Guiana	Questing ticks	30	30N	0
2	*Amblyomma calcaratum* Neumann, 1899	Kourou, Montagne des Singes, 2016, French Guiana	Engorged ticks (white-bearded Manakin [*Manacus manacus*])	1	1L	0
3	*Amblyomma coelebs* Neumann, 1899	Petit-Saut, Montagne Plomb, 2016, French Guiana	Questing ticks	10	10N	0
		Kaw, Montagne de Kaw, 2016, French Guiana	Questing ticks	1	1N	0
		Sinnamary, Piste Saint-Elie, 2016, French Guiana	Questing ticks	1	1N	0
		Kourou, Centre Spatial Guyanais, 2016, French Guiana	Questing ticks	1	1N	0
		Sinnamary, Crique Verte, 2016, French Guiana	Questing ticks	1	1N	0
4	*Amblyomma dissimile* Koch, 1884	Awala-Yalimapo, Ayawande, 2016, French Guiana	Engorged ticks (cane toad [*Rhinella marina*])	4	2N, 2M	0
		Rémire-Montjoly, 2017-2018, French Guiana	Engorged ticks (green Iguana [*Iguana iguana*])	16	10M, 6F	0
		Talhuen, Village, 2016, French Guiana	Questing ticks	2	2F	0
5	*Amblyomma geayi* Neumann, 1899	Cayenne, Montagne Tigre, 2017, French Guiana	Questing ticks	4	4L	0
		Rémire-Montjoly, Rorota, 2014-2016, French Guiana	Engorged ticks (Crimson-hooded Manakin [Pipra aureola], woodcreeper [Glyphoryncus spirurus])	2	2L	0
		Rémire-Montjoly, Vidal, 2016, French Guiana	Engorged ticks (sloth [*Bradypus tridactylus*])	2	2M	0
		Saint-Laurent-du-Maroni, Piste Voltaire, 2013, French Guiana	Engorged ticks (white-bearded Manakin [*Manacus manacus*], white-necked Thrush [*Turdus albicollis*])	2	2L	0
6	*Amblyomma goeldii* Neumann, 1899	Régina, N1 road PK63, 2017, French Guiana	Engorged ticks (tamandua [*Tamandua tetradactyla*])	5	2M, 3F	0
7	*Amblyomma humerale* Koch, 1844	Kourou, Montagne des Singes, 2016, French Guiana	Questing ticks	1	1N	0
		Station des Nouragues, 2017, French Guiana	Questing ticks	5	5M	0
		Matoury, RN, 2016, French Guiana	Engorged ticks (Gray Four-eyed Opossum [Philander opossum])	3	3N	0
		Mana, Trinité, 2017, French Guiana	Questing ticks	1	1F	0
8	*Amblyomma latepunctatum* Tonelli-Rondelli, 1939	Saint-Georges, Crique Gabaret 2016, French Guiana	Questing ticks	1	1N	0
		Petit-Saut, Montagne Plomb, 2016, French Guiana	Questing ticks	3	3N	0
9	*Amblyomma longirostre* (Koch, 1844)	Montsinéry, Bagne des Annamites, 2014, French Guiana	Engorged ticks (McConnell's flycatcher [*Mionectes macconnelli*])	2	2L	0
		Macouria, lotissement Maillard, 2012-2014, French Guiana	Engorged ticks (woodcreeper [Glyphoryncus spirurus], Crimson-hooded Manakin [Pipra aureola], blue-backed manakin [Chiroxiphia pareola])	27	26L, 1N	0
		Kourou, Montagne des Pères, 2014, French Guiana	Engorged ticks (blue-backed manakin [Chiroxiphia pareola], Crimson-hooded manakin [Pipra aureola])	11	10L, 1N	0
		Kourou, Montagne des Singes, 2014, French Guiana	Engorged ticks (woodcreeper [*Glyphoryncus spirurus*], white-bearded manakin [*Manacus manacus*], white-flanked antwren [*Myrmotherula axillaris*] golden-headed manakin [*Pipra erythrocephala*])	5	4L, 1N	0
		Rémire-Montjoly, Sentier du Rorota, 2014-2016, French Guiana	Engorged ticks (Crimson-hooded manakin [Pipra aureola], woodcreeper [Glyphoryncus spirurus], barred antshrike [Thamnophilus doliatus], buff-throated saltator [Saltator maximus], silver-beaked tanager [Ramphocelus carbo], yellow-olive flatbill [Tolmomyias sulphurescens], straight-billed woodcreeper [Dendroplex picus], golden-spangled piculet [Picumnus exilis])	54	54L	0
		Matoury, Mont Paramana, 2014 , French Guiana	Engorged ticks (Crimson-hooded manakin [Pipra aureola], white-bearded manakin [Manacus manacus])	14	13L, 1N	0
		Saint-Laurent-du-Maroni, Piste Voltaire, 2013, French Guiana	Engorged ticks (grey-breasted sabrewing [*Campylopterus largipennis*])	1	1L	0
		Matoury, PK7, 2012, French Guiana	Engorged ticks (white-bearded Manakin [*Manacus manacus*])	1	1L	0
		Montsinéry, Chemin de Risquetout, 2014, French Guiana	Engorged ticks (McConnell's flycatcher [*Mionectes macconnelli*], white-necked thrush [*Turdus albicollis*])	2	1L, 1N	0
		Saint-Laurent-du-Maroni, Saint-Jean, 2013, French Guiana	Engorged ticks (McConnell's flycatcher [*Mionectes macconnelli*], woodcreeper [*Glyphoryncus spirurus*])	2	2L	0
		Rémire-Montjoly, Vidal, 2013, French Guiana	Engorged ticks (woodcreeper [Glyphoryncus spirurus], blue-backed manakin [Chiroxiphia pareola], Crimson-hooded manakin [Pipra aureola])	17	16L, 1N	0
10	*Amblyomma naponense* (Packard, 1869)	Kaw, Montagne de Kaw, 2016, French Guiana	Questing ticks	3	3N	0
		Saint-Georges, crique Gabaret, 2016, French Guiana	Questing ticks	1	1M	0
		Mana, Trinité, 2017, French Guiana	Questing ticks	1	1F	0
11	*Amblyomma oblongoguttatum* Koch, 1844	Kourou, Centre Spatial Guyanais, 2016, French Guiana	Questing ticks	66	36M, 30F	1 (1.5%)
12	*Amblyomma pacae* Aragão, 1911	Saint-Georges, crique Gabaret, 2016, French Guiana	Questing ticks	5	5L	0
13	*Amblyomma romitii* Tonelli-Rondelli, 1939	Macouria, Tonate, 2017, French Guiana	Engorged ticks (capybara [*Hydrochoerus hydrochaeris*])	2	2F	0

14	*Amblyomma rotundatum* Koch, 1844	Iracoubo, Counamie, 2017, French Guiana	Engorged ticks (red-footed tortoise [*Chelonoidis carbonaria*])	1	1F	0
		Kourou, Montagne des Singes, 2016, French Guiana	Questing ticks	5	5L	0
15	*Amblyomma scalpturatum* Neumann, 1906	Petit-Saut, montagne Plomb, 2016, French Guiana	Questing ticks	1	1M	0
		Kourou, Centre Spatial Guyanais, 2016, French Guiana	Questing ticks	3	3N	0
		Kourou, Montagne des Singes, 2016, French Guiana	Questing ticks	1	1F	0
		Station des Nouragues, 2017, French Guiana	Questing ticks	3	3N	0
		Régina, Savane-Roche Virginie, 2016, French Guiana	Questing ticks	1	1F	0
16	*Amblyomma varium* Koch, 1844	Rémire-Montjloy, Sentier Loyola, 2017, French Guiana	Questing ticks	1	1N	0
		Matoury, Piste de la Mirande, 2016, French Guiana	Questing ticks	3	3L	0
		Matoury, Larivot, 2016, French Guiana	Engorged ticks (sloth [*Bradypus tridactylus*])	2	2M	0
		Rémire-Montjoly, Vidal, 2016, French Guiana	Engorged ticks (sloth [*Bradypus tridactylus*])	1	1F	0
17	*Dermacentor nitens* Neumann, 1897	Macouria, N1 road PK 34, 2016, French Guiana	Engorged ticks (horses [*Equus caballus*])	97	27L, 32N, 20M, 18F	0
18 -	*Haemaphysalis juxtakochi* Cooley, 1946	Kourou, Montagne des Singes, 2016, French Guiana	Questing ticks	2	2N	0
		Petit-Saut, montagne Plomb, 2016, French Guiana	Questing ticks	5	5N	0
		Montagne Favard, 2016, French Guiana	Questing tick	1	1N	0
19	*Ixodes luciae* Senevet, 1940	Cayenne, Camp du tigre, 2017, French Guiana	Engorged ticks (Gray four-eyed opossum [Philander opossum], Linnaeus's mouse opossum [Marmosa murina])	2	1L, 1M	0
		Cayenne, Montagne Tigre, 2017, French Guiana	Engorged ticks (Gray four-eyed opossum [Philander opossum])	3	1M, 1F	0
		Rémire-Montjoly, Vidal, 2016, French Guiana	Engorged ticks (large-headed rice rat [*Hylaeamys megacephalus*], Gray four-eyed opossum [*Philander opossum*])	1	2N	0
20	*Ornithodoros capensis*sensus stricto Neumann, 1901	Grand Connétable island, 2016, French Guiana	Engorged ticks (laughing gull nest (*Leucophaeus atricilla*])	6	2N, 2M, 2F	0
21	*Rhipicephalus microplus* (Canestrini, 1888)	Macouria, Matiti, 2016, French Guiana	Engorged ticks (cattle [*Bos taurus indicus*])	10	4L, 6M	0
22	*Rhipicephalus sanguineus* sensus lato (Latreille, 1806)	Kourou, rue Maurice Ravel, 2016, French Guiana	Engorged ticks (dog [*Canis lupus familiaris*])	3	3F	0
		Rémire-Montjoly, Montravel, 2016, French Guiana	Engorged ticks (dog [*Canis lupus familiaris*])	3	3M	0
Europe (France and The Netherlands)						
23	*Dermacentor marginatus* (Sulzer, 1776)	Pompignan, 2019-2021, France	Questing ticks and engorged ticks (horses [*Equus caballus*])	53	16M, 37F	0
		La Louvière-Lauragais, 2019-2020, France	Questing ticks and engorged ticks (horses [*Equus caballus*])	25	6M, 19F	0
		Longuiers, 2020, France	Questing ticks and engorged ticks (horses [*Equus caballus*])	1	1F	0
		Corse, 2019, France	Questing tick	1	1M	0
		Saint-Léon, 2020, France	Questing ticks and engorged ticks (horses [*Equus caballus*])	20	9M, 11F	0
		Seyre, 2019-2020, France	Questing ticks and engorged ticks (horses [*Equus caballus*])	3	3F	0
		Versant A, Bissy-sur-Fley, 2021, France	Questing ticks	20	10M, 10F	0
		Versant B, Bissy-sur-Fley, 2021, France	Questing ticks	20	10M, 10F	0
		Centre équestre, Hérault, 2020-2021, France	Questing ticks	5	2M, 3F	0
24	*Dermacentor reticulatus* (Fabricius, 1794)	Forêt du Herrenwald 2020-2021, France	Questing ticks	40	18M, 22F	0
		Chizé, 2020, France	Questing ticks	4	4F	0
		La Louvière-Lauragais, 2020, France	Questing ticks	3	3M	0
		Seyre, 2020, France	Questing ticks	2	2F	0
		Bernardswiller, 2020, France	Questing ticks	16	10M, 6F	0
		Gardouch, 2020, France	Engorged tick (roe deer [*Capreolus capreolus*])	1	1F	0
		Alsace, France	Questing ticks	20	20F	0
25	*Ixodes frontalis* (Panzer, 1798)	2019, France	Engorged tick (undetermined passerine species)	1	1F	0
		Leg CVFSE Philippe Gourlay, 2020, France	Engorged tick (undetermined passerine species)	3	3F	0
		St-Bauzille-de-Montmel, 2021, France	Engorged tick (captive grey parrot [*Psittacus erithacus*])	1	1F	0
		Saint Père en Retz, 2021, France	Engorged tick (undetermined passerine species)	1	1F	0
		Saint-Herblain, lieu-dit Bagatelle, 2021, France	Questing ticks	4	4N	0
		Carquefou, 2021, France	Engorged tick (undetermined passerine species)	1	1F	0
26	*Ixodes hexagonus* Leach, 1815	Hérault, France, 2019-2020, France	Engorged ticks (european hedgehog [*Erinaceus europaeus*])	3	3F	0
		St Mathieu de Tréviers, 2020, France	Engorged ticks (european hedgehog [*Erinaceus europaeus*])	1	1F	0
27	*Ixodes ricinus* (Linnaeus, 1758)	Gardouch, 2014-2021, France	Engorged ticks (roe deer [*Capreolus capreolus*])	53	8N, 6M, 39F	1 (1.9%)
		Site Vallons et Coteaux de Gascogne, 2020, France	Questing ticks and engorged ticks (roe deer [*Capreolus capreolus*])	17	3M, 14F	2 (11.8%)
		Hiesse, 2019, France	Questing ticks	1	1F	0
		Tonnay-Charente, 2019, France	Questing ticks	7	7F	0
		Chizé, 2020, France	Questing ticks and engorged ticks (roe deer [*Capreolus capreolus*])	31	18N, 3M, 10F	4 (13%)
		Forêt du Herrenwald, 2020, France	Questing ticks	30	30M	0
		Bissy-sur-Fley, 2021, France	Questing ticks	2	2F	0
		De Dorschkamp, Wageningen, 2020, The Netherlands	Questing ticks	37	19M, 18F	0
28	*Ixodes ventalloi* Gil Collado, 1936	Bretagne, 2018, France	Engorged ticks (European rabbit [*Oryctolagus cuniculus*])	10	10F	0

Table S2 Primers used in polymerase chain reaction (PCR) assays for trypanosomes screening survey and molecular typing.

Gene	Product	Primers (5'-3')		Tm	Fragment size	Reference for primer
18S rRNA	Small ribosomal subunit (SSU)	SLF	GCTTGTTTCAAGGACTTAGC	52°C	1st round PCR: SLF/S762: 1,900-2,100bp	[1], this study
		S762	GACTTTTGCTTCCTCTAATG		2nd round PCR (for screening survey): TRYP_18S_F/TRYP_18S_R: 370-400bp	
		TRYP_18S_F	AATAGAGCCGGCAGTGCC		2nd round PCR (for 18S rRNA sequencing, fragment 1): SLF/TRYP_18S_R: 900-1,000bp	
		TRYP_18S_R	CTCTGACGCACCAATACGTTC		2nd round PCR (for 18S rRNA sequencing, fragment 2): TRYP_18S_F/S762: 1,500-1,600bp	
EF1-α	Elongation factor 1-alpha	Tryp_EF1a_F	AGCAGATGGTYGTSTGCTGCA	52°C	1st round PCR: Tryp_EF1a_F/Tryp_EF1a_R2: 762bp	This study
		Tryp_EF1a_R1	ATCACCTGYGCCGTGAAGTCGGC		2nd round PCR: Tryp_EF1a_F/Tryp_EF1a_R2: 562bp	
		Tryp_EF1a_R2	ACCTCCACGCACATRGGCTTCTG			
hsp60	60-kDa heat shock protein	Tryp_Hsp60_F1	GAGAACATTGTGCAGGTKGCCAC	52°C	1st round PCR: Tryp_Hsp60_F1/ Tryp_Hsp60_R2: 918bp	This study
		Tryp_Hsp60_F2	CAGAAGGCYGAGCTGGAGGATG		2nd round PCR: Tryp_Hsp60_F2/ Tryp_Hsp60_R1: 476bp	
		Tryp_Hsp60_R1	TGCAGCTTYTCGCGGTTGTAGTC			
		Tryp_Hsp60_R2	CTGCACRCCGGTGCGCTGGTCCG			
hsp85	85-kDa heat shock protein	Tryp_HS85_F	AGATCCGSTACCAGAGCCTGAC	52°C	1st round PCR: Tryp_Hsp85_F/ Tryp_Hsp85_R2: 1,026bp	This study
		Tryp_HS85_R1	CCAGTCGTTCGAGATGGCCTTG		2nd round PCR: Tryp_Hsp85_F/ Tryp_Hsp85_R1: 767bp	
		Tryp_HS85_R2	TGTTCTGCTGCAGGTTCTCAC			

Figure legends

Fig. S1 Phylogenies of the *Trypanosoma pestanai* clade constructed using maximum-likelihood (ML) estimations based on the 5' end region of the 18S rRNA gene sequence (241 unambiguously aligned bp; best-fit approximation for the evolutionary model: GTR). *Trypanosoma* sequences obtained in this study are shown in bold. Origin of the *Trypanosoma* sequences (*i.e.,* from ticks, mammals either marsupials or placentals) is shown by black squares, while host species and geographic origin are listed next to the name of *Trypanosoma* species or strain. GenBank accession numbers of sequences used in analyses are shown on the phylogenetic trees. Numbers at nodes indicate percentage support of 1,000 bootstrap replicates. Only bootstrap values >70% are shown.

Fig. S2 Phylogenies of the *Trypanosoma pestanai* clade constructed using maximum-likelihood (ML) estimations based on the middle region of the 18S rRNA gene sequence (276 unambiguously aligned bp; best-fit approximation for the evolutionary model: GTR). *Trypanosoma* sequences obtained in this study are shown in bold. Origin of the *Trypanosoma* sequences (*i.e.,* from ticks, mammals either marsupials or placentals) is shown by black squares, while host species and geographic origin are listed next to the name of *Trypanosoma* species or strain. GenBank accession numbers of sequences used in analyses are shown on the phylogenetic trees. Numbers at nodes indicate percentage support of 1,000 bootstrap replicates. Only bootstrap values >70% are shown.

Fig. S3 Phylogenies of the *Trypanosoma pestanai* clade constructed using maximum-likelihood (ML) estimations based on the 3' end region of the 18S rRNA gene sequence (157 unambiguously aligned bp; best-fit approximation for the evolutionary model: GTR). *Trypanosoma* sequences obtained in this study are shown in bold. Origin of the *Trypanosoma* sequences (*i.e.,* from ticks, mammals either marsupials or placentals) is shown by black

squares, while host species and geographic origin are listed next to the name of *Trypanosoma* species or strain. GenBank accession numbers of sequences used in analyses are shown on the phylogenetic trees. Numbers at nodes indicate percentage support of 1,000 bootstrap replicates. Only bootstrap values >70% are shown.

Fig. S4 Phylogenies of trypanosomes constructed using maximum-likelihood (ML) estimations based on the 18S rRNA gene sequence (237 unambiguously aligned bp; best-fit approximation for the evolutionary model: GTR). Black arrows indicate infection of the Australian tick *A. triguttatum* by *T. noyesi* which belongs to the *T. cruzi* clade [2]. GenBank accession numbers of sequences used in analyses are shown on the phylogenetic trees. Numbers at nodes indicate percentage support of 1,000 bootstrap replicates. Only bootstrap values >70% are shown.

Fig. S5 Phylogenies of trypanosomes constructed using maximum-likelihood (ML) estimations based on the 18S rRNA gene sequence (203 unambiguously aligned bp; best-fit approximation for the evolutionary model: GTR). Black arrow indicates infections of the Australian ticks *I. tasmani* and *I. holocyclus* by *T. irwini* which belongs to the *T. irwini* clade [3]. GenBank accession numbers of sequences used in analyses are shown on the phylogenetic trees. Numbers at nodes indicate percentage support of 1,000 bootstrap replicates. Only bootstrap values >70% are shown.

References

1. McInnes LM, Gillett A, Ryan UM, Austen J, Campbell RSF, Hanger J, et al. *Trypanosoma irwini* n. sp (Sarcomastigophora: Trypanosomatidae) from the koala (*Phascolarctos cinereus*). Parasitology. 2009;136:875–85.

2. Krige AS, Thompson RCA, Seidlitz A, Keatley S, Botero A, Clode PL. "Hook, line, and sinker": Fluorescence in situ hybridisation (FISH) uncovers *Trypanosoma noyesi* in Australian questing ticks. Ticks Tick Borne Dis. 2021;12.

3. Barbosa AD, Gofton AW, Paparini A, Codello A, Greay T, Gillett A, et al. Increased genetic diversity and prevalence of co-infection with *Trypanosoma spp.* in koalas (*Phascolarctos cinereus*) and their ticks identified using next-generation sequencing (NGS). PLoS One. 2017;12.

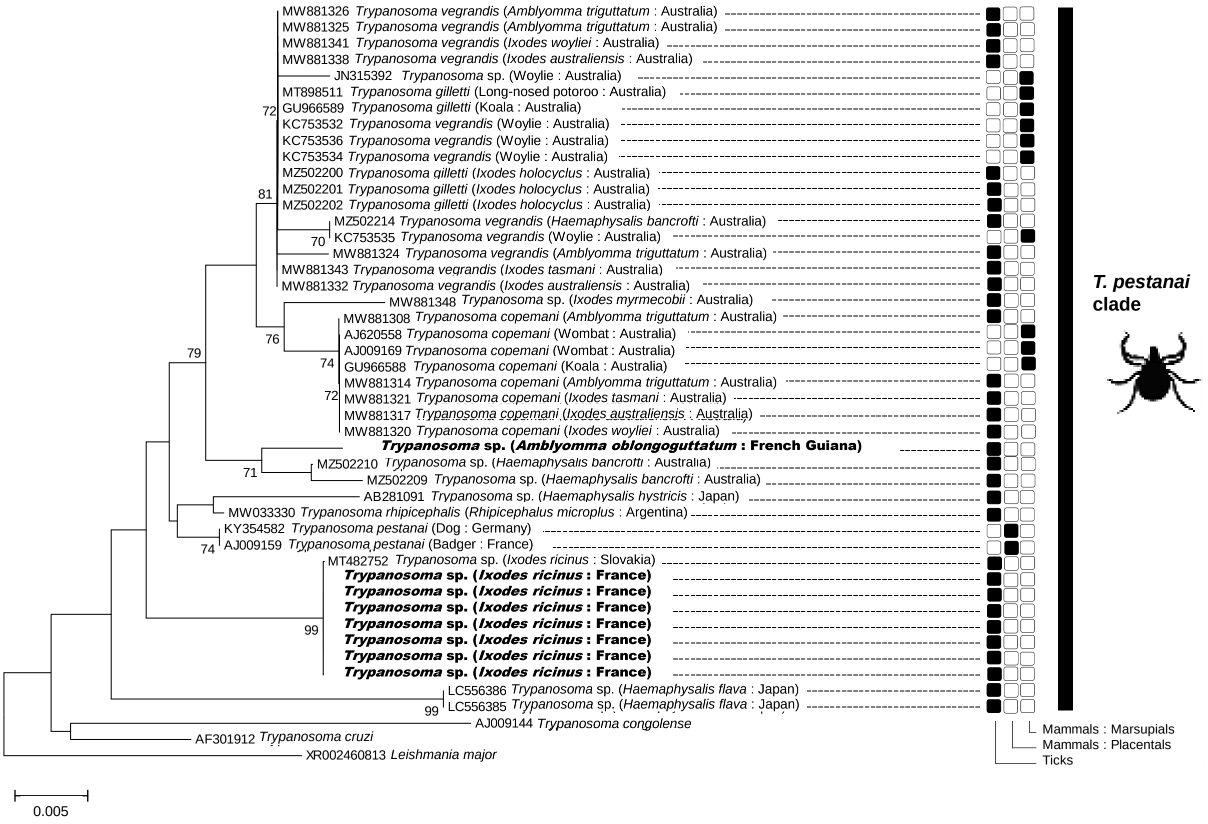

MW881326 Trypanosoma vegrandis (Amblyomma triguttatum : Australia)
MW881325 Trypanosoma vegrandis (Amblyomma triguttatum : Australia)
MW881341 Trypanosoma vegrandis (Ixodes woyliei : Australia)
MW881338 Trypanosoma vegrandis (Ixodes australiensis : Australia)
JN315392 Trypanosoma sp. (Woylie : Australia)
MT898511 Trypanosoma gilletti (Long-nosed potoroo : Australia)
GU966589 Trypanosoma gilletti (Koala : Australia)
KC753532 Trypanosoma vegrandis (Woylie : Australia)
KC753536 Trypanosoma vegrandis (Woylie : Australia)
KC753534 Trypanosoma vegrandis (Woylie : Australia)
MZ502200 Trypanosoma gilletti (Ixodes holocyclus : Australia)
MZ502201 Trypanosoma gilletti (Ixodes holocyclus : Australia)
MZ502202 Trypanosoma gilletti (Ixodes holocyclus : Australia)
MZ502214 Trypanosoma vegrandis (Haemaphysalis bancrofti : Australia)
KC753535 Trypanosoma vegrandis (Woylie : Australia)
MW881324 Trypanosoma vegrandis (Amblyomma triguttatum : Australia)
MW881343 Trypanosoma vegrandis (Ixodes tasmani : Australia)
MW881332 Trypanosoma vegrandis (Ixodes australiensis : Australia)
MW881348 Trypanosoma sp. (Ixodes myrmecobii : Australia)
MW881308 Trypanosoma copemani (Amblyomma triguttatum : Australia)
AJ620558 Trypanosoma copemani (Wombat : Australia)
AJ009169 Trypanosoma copemani (Wombat : Australia)
GU966588 Trypanosoma copemani (Koala : Australia)
MW881314 Trypanosoma copemani (Amblyomma triguttatum : Australia)
MW881321 Trypanosoma copemani (Ixodes tasmani : Australia)
MW881317 Trypanosoma copemani (Ixodes australiensis : Australia)
MW881320 Trypanosoma copemani (Ixodes woyliei : Australia)
Trypanosoma sp. (Amblyomma oblongoguttatum : French Guiana)
MZ502210 Trypanosoma sp. (Haemaphysalis bancrofti : Australia)
MZ502209 Trypanosoma sp. (Haemaphysalis bancrofti : Australia)
AB281091 Trypanosoma sp. (Haemaphysalis hystricis : Japan)
MW033330 Trypanosoma rhipicephalis (Rhipicephalus microplus : Argentina)
KY354582 Trypanosoma pestanai (Dog : Germany)
AJ009159 Trypanosoma pestanai (Badger : France)
MT482752 Trypanosoma sp. (Ixodes ricinus : Slovakia)
Trypanosoma sp. (Ixodes ricinus : France)
Trypanosoma sp. (Ixodes ricinus : France)
Trypanosoma sp. (Ixodes ricinus : France)
Trypanosoma sp. (Ixodes ricinus : France)
Trypanosoma sp. (Ixodes ricinus : France)
Trypanosoma sp. (Ixodes ricinus : France)
Trypanosoma sp. (Ixodes ricinus : France)
LC556386 Trypanosoma sp. (Haemaphysalis flava : Japan)
LC556385 Trypanosoma sp. (Haemaphysalis flava : Japan)
AJ009144 Trypanosoma congolense
AF301912 Trypanosoma cruzi
XR002460813 Leishmania major
72
81
70
76
79
74
72
71
74
99
99
T. pestanai clade
Mammals : Marsupials
Mammals : Placentals
Ticks
0.005

Figure S1

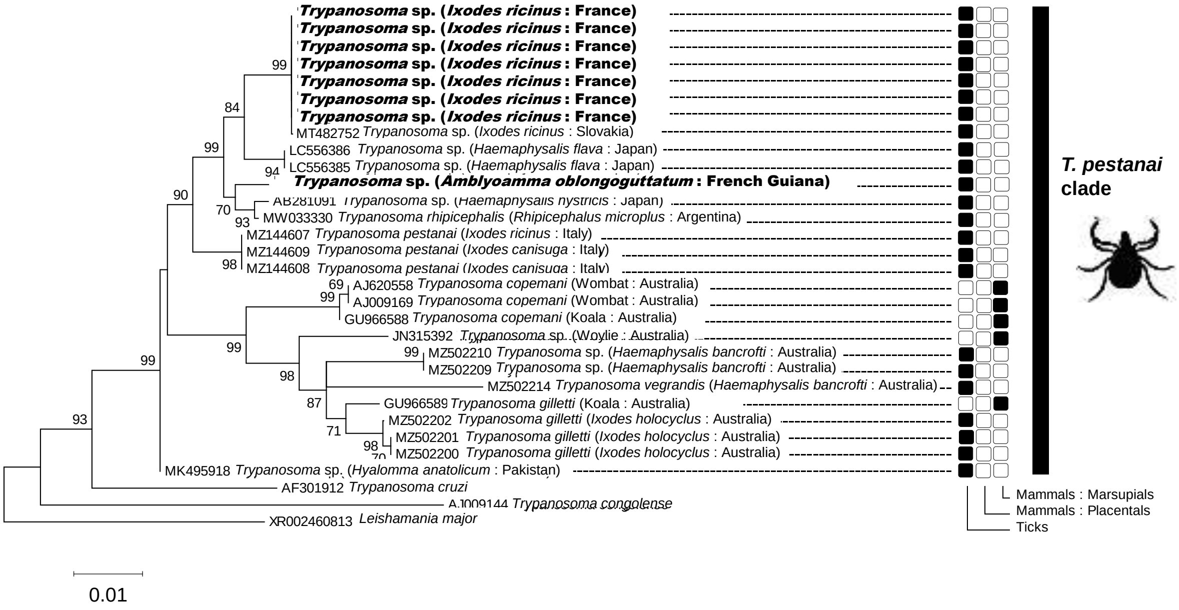

Trypanosoma sp. (Ixodes ricinus : France)
Trypanosoma sp. (Ixodes ricinus : France)
Trypanosoma sp. (Ixodes ricinus : France)
Trypanosoma sp. (Ixodes ricinus : France)
Trypanosoma sp. (Ixodes ricinus : France)
Trypanosoma sp. (Ixodes ricinus : France)
Trypanosoma sp. (Ixodes ricinus : France)
MT482752 Trypanosoma sp. (Ixodes ricinus : Slovakia)
LC556386 Trypanosoma sp. (Haemaphysalis flava : Japan)
LC556385 Trypanosoma sp. (Haemaphysalis flava : Japan)
Trypanosoma sp. (Amblyoamma oblongoguttatum : French Guiana)
AB281091 Trypanosoma sp. (Haemaphysalis hystricis : Japan)
MW033330 Trypanosoma rhipicephalis (Rhipicephalus microplus : Argentina)
MZ144607 Trypanosoma pestanai (Ixodes ricinus : Italy)
MZ144609 Trypanosoma pestanai (Ixodes canisuga : Italy)
MZ144608 Trypanosoma pestanai (Ixodes canisuga : Italy)
AJ620558 Trypanosoma copemani (Wombat : Australia)
AJ009169 Trypanosoma copemani (Wombat : Australia)
GU966588 Trypanosoma copemani (Koala : Australia)
JN315392 Trypanosoma sp. (Woylie : Australia)
MZ502210 Trypanosoma sp. (Haemaphysalis bancrofti : Australia)
MZ502209 Trypanosoma sp. (Haemaphysalis bancrofti : Australia)
MZ502214 Trypanosoma vegrandis (Haemaphysalis bancrofti : Australia)
GU966589 Trypanosoma gilletti (Koala : Australia)
MZ502202 Trypanosoma gilletti (Ixodes holocyclus : Australia)
MZ502201 Trypanosoma gilletti (Ixodes holocyclus : Australia)
MZ502200 Trypanosoma gilletti (Ixodes holocyclus : Australia)
MK495918 Trypanosoma sp. (Hyalomma anatolicum : Pakistan)
AF301912 Trypanosoma cruzi
AJ009144 Trypanosoma congolense
XR002460813 Leishamania major
99
84
99
94
90
70
93
98
69
99
99
99
98
87
71
98
70
99
93
T. pestanai clade
Mammals : Marsupials
Mammals : Placentals
Ticks
0.01

Figure S2

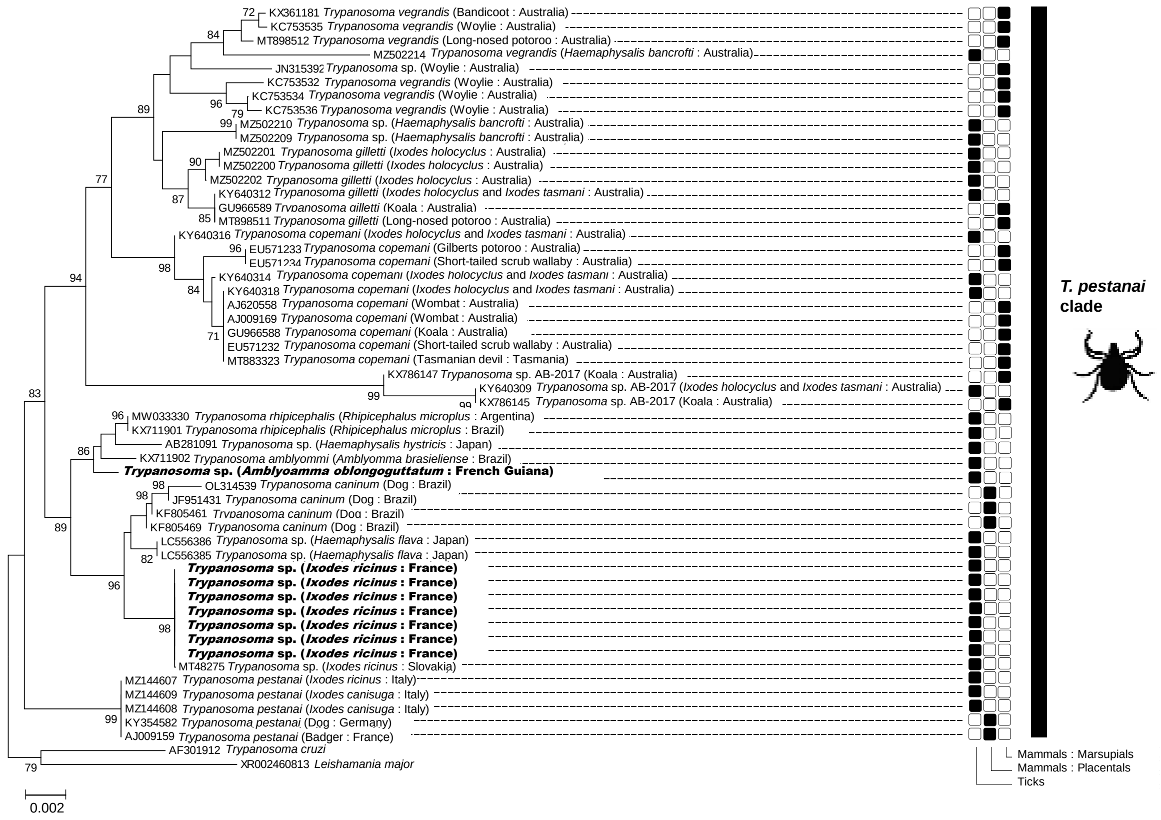
T. pestanai clade
Mammals : Marsupials
Mammals : Placentals
Ticks
0.002

Figure S3

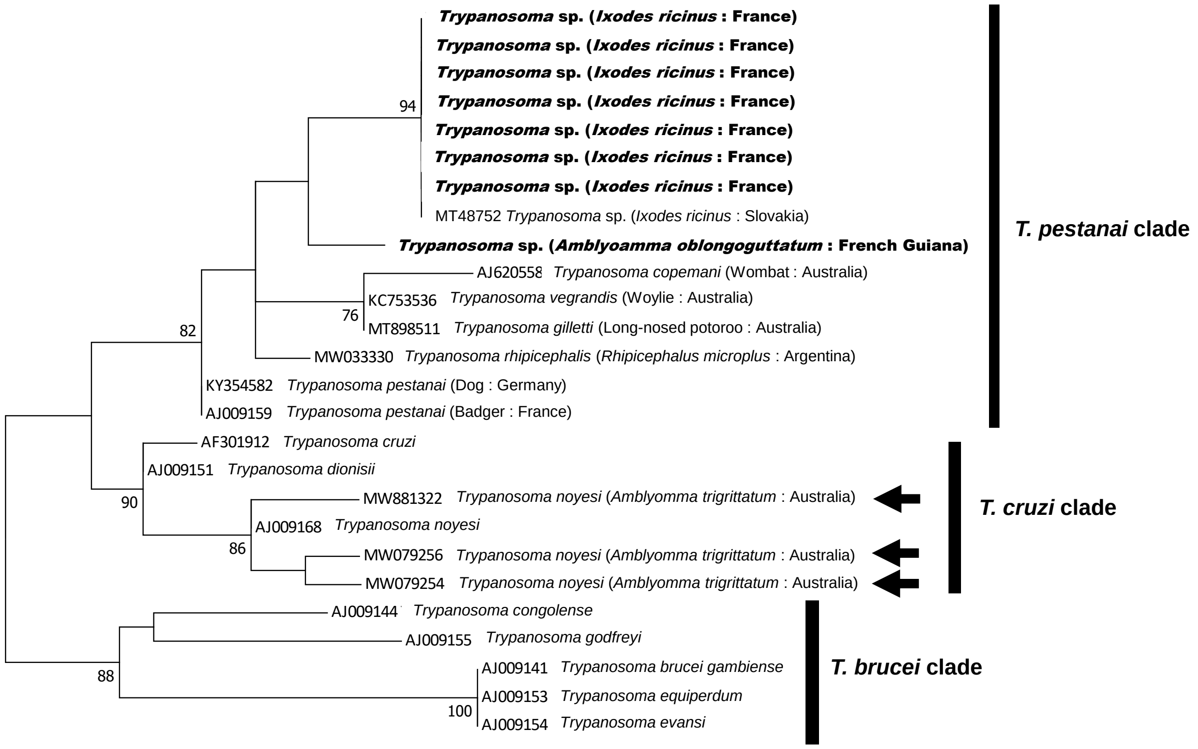
Trypanosoma sp. (Ixodes ricinus : France)
Trypanosoma sp. (Ixodes ricinus : France)
Trypanosoma sp. (Ixodes ricinus : France)
94
Trypanosoma sp. (Ixodes ricinus : France)
Trypanosoma sp. (Ixodes ricinus : France)
Trypanosoma sp. (Ixodes ricinus : France)
Trypanosoma sp. (Ixodes ricinus : France)
MT48752 Trypanosoma sp. (Ixodes ricinus : Slovakia)
Trypanosoma sp. (Amblyoamma oblongoguttatum : French Guiana)
AJ620558 Trypanosoma copemani (Wombat : Australia)
KC753536 Trypanosoma vegrandis (Woylie : Australia)
76
MT898511 Trypanosoma gilletti (Long-nosed potoroo : Australia)
82
MW033330 Trypanosoma rhipicephalis (Rhipicephalus microplus : Argentina)
KY354582 Trypanosoma pestanai (Dog : Germany)
AJ009159 Trypanosoma pestanai (Badger : France)
T. pestanai clade
AF301912 Trypanosoma cruzi
AJ009151 Trypanosoma dionisii
90
MW881322 Trypanosoma noyesi (Amblyomma trigrittatum : Australia)
AJ009168 Trypanosoma noyesi
86
MW079256 Trypanosoma noyesi (Amblyomma trigrittatum : Australia)
MW079254 Trypanosoma noyesi (Amblyomma trigrittatum : Australia)
T. cruzi clade
AJ009144 Trypanosoma congolense
AJ009155 Trypanosoma godfreyi
88
AJ009141 Trypanosoma brucei gambiense
AJ009153 Trypanosoma equiperdum
100
AJ009154 Trypanosoma evansi
T. brucei clade

Figure S4

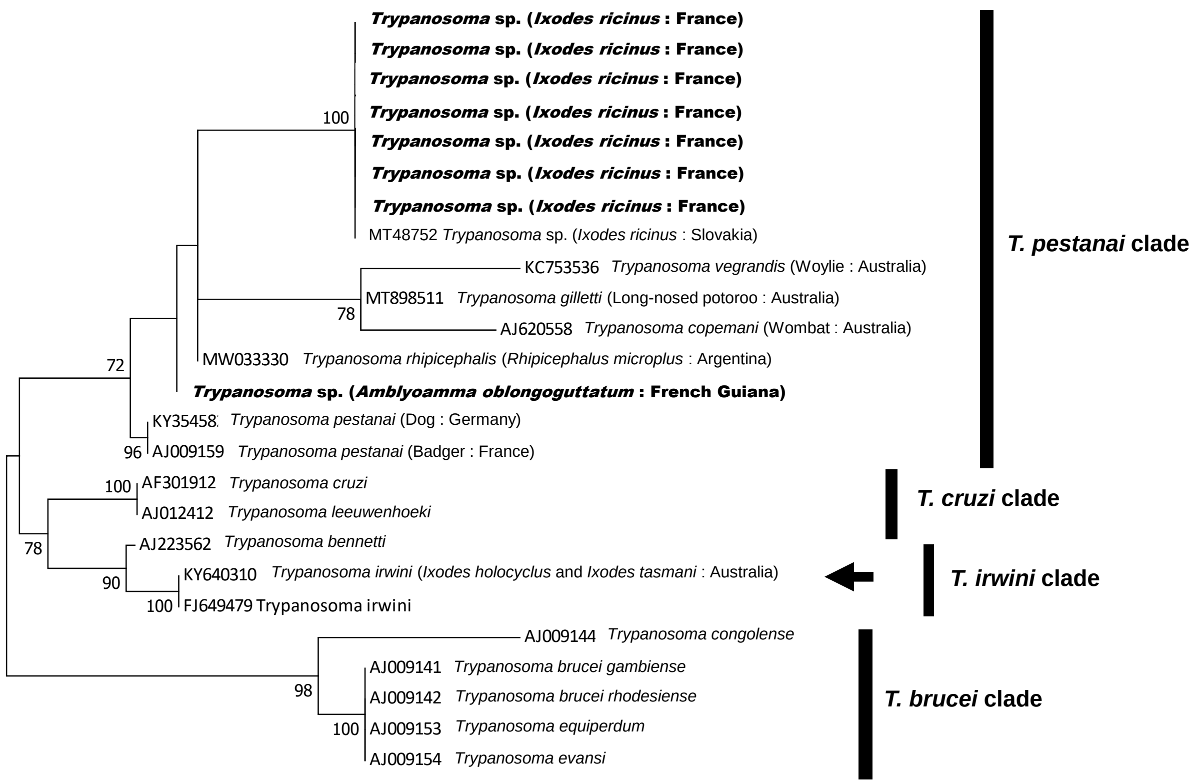
Trypanosoma sp. (Ixodes ricinus : France)
Trypanosoma sp. (Ixodes ricinus : France)
Trypanosoma sp. (Ixodes ricinus : France)
Trypanosoma sp. (Ixodes ricinus : France)
Trypanosoma sp. (Ixodes ricinus : France)
Trypanosoma sp. (Ixodes ricinus : France)
Trypanosoma sp. (Ixodes ricinus : France)
100
MT48752 Trypanosoma sp. (Ixodes ricinus : Slovakia)
KC753536 Trypanosoma vegrandis (Woylie : Australia)
MT898511 Trypanosoma gilletti (Long-nosed potoroo : Australia)
78
AJ620558 Trypanosoma copemani (Wombat : Australia)
MW033330 Trypanosoma rhipicephalis (Rhipicephalus microplus : Argentina)
72
Trypanosoma sp. (Amblyoamma oblongoguttatum : French Guiana)
KY35458 Trypanosoma pestanai (Dog : Germany)
96
AJ009159 Trypanosoma pestanai (Badger : France)
100
AF301912 Trypanosoma cruzi
AJ012412 Trypanosoma leeuwenhoeki
78
AJ223562 Trypanosoma bennetti
90
KY640310 Trypanosoma irwini (Ixodes holocyclus and Ixodes tasmani : Australia)
100
FJ649479 Trypanosoma irwini
AJ009144 Trypanosoma congolense
98
AJ009141 Trypanosoma brucei gambiense
AJ009142 Trypanosoma brucei rhodesiense
100
AJ009153 Trypanosoma equiperdum
AJ009154 Trypanosoma evansi
T. pestanai clade
T. cruzi clade
T. irwini clade
T. brucei clade

Figure S5
